# miR319, miR390, and miR393 Are Involved in Aluminum Response in Flax (*Linum usitatissimum* L.)

**DOI:** 10.1155/2017/4975146

**Published:** 2017-02-19

**Authors:** Alexey A. Dmitriev, Anna V. Kudryavtseva, Nadezhda L. Bolsheva, Alexander V. Zyablitsin, Tatiana A. Rozhmina, Natalya V. Kishlyan, George S. Krasnov, Anna S. Speranskaya, Anastasia A. Krinitsina, Asiya F. Sadritdinova, Anastasiya V. Snezhkina, Maria S. Fedorova, Olga Yu. Yurkevich, Olga V. Muravenko, Maxim S. Belenikin, Nataliya V. Melnikova

**Affiliations:** ^1^Engelhardt Institute of Molecular Biology, Russian Academy of Sciences, Moscow 119991, Russia; ^2^All-Russian Research Institute for Flax, Torzhok 172002, Russia; ^3^Faculty of Biology, Lomonosov Moscow State University, Moscow 119991, Russia

## Abstract

Acid soils limit agricultural production worldwide. Major reason of crop losses in acid soils is the toxicity of aluminum (Al). In the present work, we investigated expression alterations of microRNAs in flax (*Linum usitatissimum* L.) plants under Al stress. Flax seedlings of resistant (TMP1919 and G1071/4_k) and sensitive (Lira and G1071/4_o) to Al cultivars and lines were exposed to AlCl_3_ solution for 4 and 24 hours. Twelve small RNA libraries were constructed and sequenced using Illumina platform. In total, 97 microRNAs from 18 conserved families were identified. miR319, miR390, and miR393 revealed expression alterations associated with Al treatment of flax plants. Moreover, for miR390 and miR393, the alterations were distinct in sensitive and resistant to Al genotypes. Expression level changes of miR319 and miR390 were confirmed using qPCR analysis. In flax, potential targets of miR319 are TCPs, miR390–TAS3 and GRF5, and miR393–AFB2-coding transcripts. TCPs, TAS3, GRF5, and AFB2 participate in regulation of plant growth and development. The involvement of miR319, miR390, and miR393 in response to Al stress in flax was shown here for the first time. We speculate that these microRNAs play an important role in Al response* via* regulation of growth processes in flax plants.

## 1. Introduction

Acid soils result in decrease of agricultural production all over the world [1]. Toxicity of aluminum (Al) is a major reason of crop losses in acid soils [2]. Different mechanisms of plant response to Al stress were identified: organic acid exudation by roots to chelate Al ions in soil, detoxification of Al in plants* via* chelation or transportation into the vacuole, modifications of cell wall to alter Al binding with its components, and so forth [3–5].

MicroRNA (miRNA) negatively regulates gene expression and in this way controls numerous biological processes in plants [6], including stress response [7–9]. Gene expression regulation* via* miRNA was revealed as one of the mechanisms of response to Al in different plant species [10–12]. However, there is no data on involvement of miRNAs in response to Al stress in important agricultural plant, flax (*Linum usitatissimum* L.). Flax fiber is utilized in textile industry; flax seeds are used for production of oil, linoleum, food, and pharmaceutical products [13–15]. Flax genetics and epigenetics are in the focus of research interest [16–20]. In the previous works on flax, the involvement of miRNAs in response to saline and alkaline stresses [21] and excessive or deficient nutrition [22–24] was shown.

In the present work, we performed high-throughput sequencing of flax small RNAs under normal conditions and Al exposure to identify miRNAs, whose expression was altered in response to aluminum stress, and suggested potential targets of these miRNAs in flax to speculate on affected signaling pathways.

## 2. Materials and Methods

### 2.1. Plant Material

*L. usitatissimum* plants of resistant (TMP1919 and G1071/4_k) and sensitive (Lira and G1071/4_o) to aluminum cultivars and lines were used in the present study. Seeds germinated on filter paper soaked with distilled water for 5 days. Then seedlings were transferred to falcon tubes with filter paper soaked with a 0.5 mM CaCl_2_ solution at pH 4.5 for 24 h before being exposed to a 0.5 mM CaCl_2_ solution (pH 4.5) containing 0 (N) or 500 *μ*M AlCl_3_ for 4 (Al-4) and 24 (Al-24) hours. Roots were cut off and immediately frozen in liquid nitrogen. Plant samples were stored at −70°C.

Total RNA was extracted from roots of flax plants using RNA MicroPrep kit (Zymo Research, USA). RNA quality and concentration were determined by Qubit 2.0 fluorometer (Life Technologies, USA) and Agilent 2100 Bioanalyzer (Agilent Technologies, USA). For further analysis, only RNA samples with RNA Integrity Number (RIN) value not less than 8.0 were used.

### 2.2. Flax Small RNA Sequencing

Library preparation was performed using Illumina TruSeq small RNA preparation kit (Illumina, USA) in compliance with manufacturer's protocol. Twelve libraries from pooled plant samples were obtained: N, Al-4, and Al-24 for each of four cultivars/lines (TMP1919, G1071/4_k, G1071/4_o, and Lira). Library quality was evaluated using Agilent 2100 Bioanalyzer (Agilent Technologies). The sequencing was performed on Genome Analyzer IIx (Illumina).

### 2.3. Bioinformatics Analysis of miRNAs

Low-quality reads and adapter reads were removed from raw sequencing data using Trimmomatic [25]. For further analysis, we used cleaned reads with abundance six or more. To identify conserved miRNAs in flax, small RNA sequences were aligned with known matured miRNA sequences from miRBase 21.0 [26]. Prediction of miRNA targets was performed using psRNATarget server [27] with default parameters using identified* L. usitatissimum *transcripts [16, 28].

miRNA levels were normalized to obtain reads per million (RPM) values. The comparison of miRNA expression levels in Al-4 and Al-24 libraries with N library was performed using* fold change* parameter: FC = RPM in Al-4 or Al-24/RPM in N. *P* values were calculated using *χ*^2^ test with Benjamini-Hochberg multiple testing correction. Changes were considered significant if FC or 1/FC were 1.5 or higher, that is, absolute value of log_2_⁡FC ≥ 0.58.

### 2.4. Quantitative PCR (qPCR) Analysis of miRNA Expression

We performed qPCR analysis to evaluate expression of miR319 and miR390. TaqMan MicroRNA Assays aau-miR319 and ath-miR390a (Thermo Fisher Scientific, USA) were used. Reverse transcription was performed in 15 *μ*L reaction containing 1x RT primer (Thermo Fisher Scientific), 200 U of RevertAid Reverse Transcriptase (Thermo Fisher Scientific), 1x Reverse Transcription Buffer, 250 nM of dNTPs, and 10 ng of total RNA using the following program: 16°C for 30 min, 42°C for 30 min, and 85°C for 5 min. QPCR was performed using the 7500 Real-Time PCR System in a 20 *μ*L reaction mix containing 1x PCR mix (GenLab, Russia), 250 nM of dNTPs, 2 U of polymerase (GenLab), Rox dye, and RT product using the following program: 95°C for 15 min, 40 cycles of 95°C for 15 s, and 60°C for 60 s. Three technical replicates were performed. For the evaluation of expression level alterations, ΔΔ*C*_*t*_^eff^ values, which are directly proportional to the expression level changes, were calculated [29, 30].* ETIF3H* and* ETIF3E* were chosen as the reference genes for the qPCR data analysis [24, 29, 31]. All the calculations were done using the Analysis of Transcription of Genes software [24, 32]. Correlation between high-throughput sequencing (log_2_⁡FC) and qPCR (ΔΔ*C*_*t*_^eff^) expression data was evaluated using Spearman's correlation coefficient.

## 3. Results and Discussion

Seedlings of resistant (TMP1919 and G1071/4_k) and sensitive (Lira and G1071/4_o) to Al flax cultivars and lines were exposed to Al for 4 and 24 hours. Twelve small RNA libraries were constructed and sequenced on Illumina GAIIx. In total, about 40 million raw reads were obtained. All the sequences were deposited in the European Nucleotide Archive, accession number PRJEB15342.

Search for flax miRNAs using miRBase sequences led to identification of 97 potential flax miRNAs from 18 conserved families: miR156, miR157, miR159, miR160, miR162, miR164, miR165, miR166, miR167, miR168, miR171, miR319, miR390, miR393, miR394, miR396, miR398, and miR408 (Supplementary Table  1 in Supplementary Material available online at https://doi.org/10.1155/2017/4975146). Among these miRNAs, the search for Al responsive miRNAs was performed. To reveal common trends specific to flax plants, we evaluated expression alterations after 4 and 24 hours of Al treatment using pooled data for all examined cultivars and lines (log_2_⁡FC values are represented in Table 1).

After 4 hours of Al exposure, we observed significant (absolute value of log_2_⁡FC ≥ 0.58) upregulation of miR164, miR319, miR393, and miR394 and downregulation of miR159, miR167, and miR408. After 24 hours of Al exposure, significant expression decrease was revealed for all 18 miRNA families except miR393. Thus, after 4 hours of Al treatment, miRNA levels of different families were increased, decreased, or stable. However, after 24 hours of Al exposure, expression was decreased for almost all miRNA families.

We also performed analysis of expression alterations of miRNA families in individual cultivars and lines to identify trends in resistant and sensitive to Al flax plants (Supplementary Table  2). log_2_⁡FC values are represented in Table 1. Some of the miRNAs showed opposite directions of expression alterations in studied flax cultivars and lines. As seen from Table 1, after 4 hours of Al exposure, miR156, miR157, miR162, miR165, miR166, and miR171 were significantly upregulated in one of the resistant genotypes and significantly downregulated in the other one. The same was observed for miR171 and miR408 in sensitive genotypes. We suggested that these miRNAs with opposite regulation under Al stress in resistant or sensitive cultivars and lines do not play the key role in flax response to Al. After 24 hours of Al treatment, the majority of miRNAs was downregulated in all 4 cultivars and lines.

**Figure 1:**
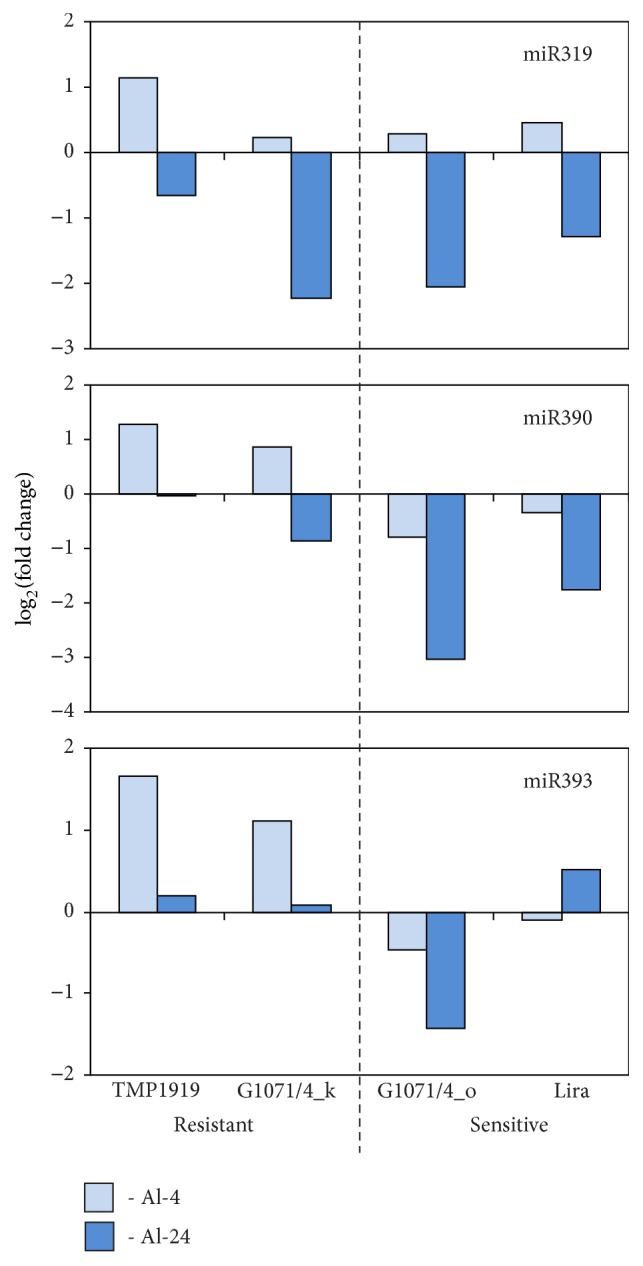
Expression alterations of miR319, miR390, and miR393 in resistant and sensitive flax cultivars and lines under Al stress.

Definite regularities were revealed for expression alterations of miR319, miR390, and miR393 families. The level of miR319 was changed in a similar way in resistant and sensitive to Al cultivars and lines: expression was increased after 4 hours of Al exposure (log_2_⁡FC varied from 0.22 to 1.16) and decreased after 24 hours (log_2_⁡FC varied from −0.65 to −2.23; Figure 1). miR390 level was decreased after 4 hours of Al exposure in sensitive to Al flax genotypes (log_2_⁡FC was −0.78 for G1071/4_o and −0.35 for Lira), but increased in resistant genotypes (log_2_⁡FC was 1.27 in TMP1919 and 0.85 in G1071/4_k; Figure 1). Moreover, after 24 hours of Al exposure, we revealed retention or moderate downregulation of miR390 in resistant to Al cultivar and line (log_2_⁡FC was −0.01 for TMP1919 and −0.84 for G1071/4_k), but strong downregulation in sensitive cultivar and line (log_2_⁡FC was −3.04 for G1071/4_o and −1.77 for Lira). miR393 was upregulated in resistant to Al genotypes (log_2_⁡FC was 1.66 for TMP1919 and 1.10 for G1071/4_k), but slightly decreased in sensitive to Al genotypes (log_2_⁡FC was −0.46 for G1071/4_o and −0.10 for Lira) after 4 hours of Al exposure (Figure 1). After 24 hours of Al exposure, miR393 level was stable in resistant to Al genotypes (log_2_⁡FC was 0.20 for TMP1919 and 0.07 for G1071/4_k) and deregulated in sensitive to Al genotypes (log_2_⁡FC was −1.43 for G1071/4_o and 0.51 for Lira).

**Figure 2:**
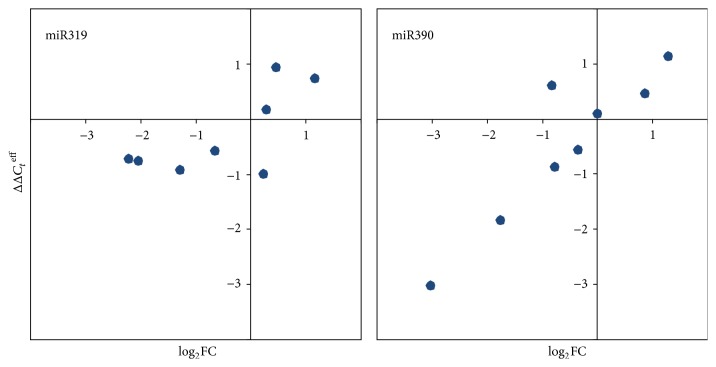
Correlation between qPCR (ΔΔ*C*_*t*_^eff^) and high-throughput sequencing (log_2_⁡FC) expression data for miR319 and miR390 in flax cultivars and lines under Al stress.

For validation of high-throughput sequencing data, expression of miR319 and miR390 was evaluated using qPCR. RNA samples of flax plants, which were used for high-throughput sequencing and were taken into qPCR analysis. The data obtained by qPCR and high-throughput sequencing methods were highly consistent: Spearman's correlation coefficient was 0.68 for miR319 and 0.76 for miR390 (*P* < 0.05; Figure 2).

In plants, miR319, miR390, and miR393 families have been identified as Al responsive [10, 11]. For miR319, both upregulation [33] and downregulation [34] were revealed in* Medicago truncatula* in response to Al stress. miR390 was upregulated in wild soybean under Al exposure [35]. However, in* M. truncatula*, miR390 was slightly downregulated after short-term Al treatment and was significantly upregulated after long-term one [34]. For miR393, upregulation was revealed in common bean roots in response to Al treatment for 24 hours [36], and downregulation in rice roots after 8 hours of Al treatment [37]. Opposite directions of the alterations of miRNA levels could be associated with different time of Al treatment or diverse resistance of examined genotypes to Al stress.

In previous flax studies, potential targets for some miRNAs from miR319, miR390, and miR393 families were predicted [21, 38, 39]. Here, we performed target prediction for flax highly-expressed miRNAs from miR319, miR390, and miR393 families (Supplementary Table  3).

For miR319, the following targets were predicted by us in flax: transcripts encoding cysteine-rich secretory proteins, antigen 5, pathogenesis-related 1 protein; myb domain protein 65 (MYB65); Teosinte Branched/Cycloidea/PCF transcription factor 3 (TCP3) superfamily protein; TCP family transcription factor 4; ABC transporter. Within predicted targets of miR319, Lus10002195 and Lus10000463 transcripts encoding TCP3 and TCP4 are the most interesting. It was previously shown that miR319 targets mRNA of TCP transcription factors, which control plant growth and development [40–42]. We suggest that the most probable target of miR319 in flax is also TCPs.

For miR390, predicted targets in flax were transcripts encoding leucine-rich receptor-like protein kinase family protein; protein kinase superfamily protein; growth-regulating factor 5 (GRF5); root hair specific 10 (RHS10); poor homologous synapsis 1 (PHS1); leucine-rich repeat (LRR) family protein; TAS3. Transcript Lus10009533 encoding GRF5 was one of the potential targets of miR390. GRFs play important role in plant developmental processes and growth under adverse environments and could be regulated by TCP4 [43]. TAS3-coding transcript, genolin_c19878 from* L. usitatissimum* unigene library, was also predicted as potential target of miR390. In other plant species, miR390 initiates tasiRNA (trans-acting small interfering RNA) biogenesis* via* cleavage or interaction with TAS3 transcript. TAS3 tasiRNAs negatively regulate ARFs (auxin response factors) that is necessary for proper plant development [44–48]. We suppose that TAS3 and GRF5 are the most likely targets of miR390 in flax.

Auxin signaling F-box 2 (AFB2), zinc finger (C3HC4-type RING finger) family protein, and pol-like 5 (PLL5) transcripts were predicted as targets of miR393 in flax. Lus10031991 and Lus10035160 encoding AFB2 are the most interesting targets of miR393. It was previously reported that targets of miR393 are AFB1 (auxin f-box protein1), AFB2, and AFB3, which are involved in auxin signaling [32, 49–51]. We speculate that miR393 probably regulates AFB2 expression in flax.

Thus, expression alterations of miR319, miR390, and miR393, which were revealed as Al-responsive in flax, could affect expression level of a number of key transcripts involved in plant growth and development.

## 4. Conclusions

High-throughput sequencing and qPCR analyses of flax small RNAs allowed us to reveal miRNAs with expression alterations under Al exposure. Our results suggest the involvement of miR319, miR390, and miR393 in Al response in* L. usitatissimum* plants. Moreover, we revealed diverse alterations of miR390 and miR393 levels in resistant and sensitive to Al genotypes. We concluded that, in flax, potential targets of miR319 are TCPs, miR390–TAS3 and GRF5, and miR393–AFB2. Thus, we speculate that miR319, miR390, and miR393 play an important role in Al stress response* via* regulation of growth and development processes in flax plants.

## Supplementary Material

MicroRNAs in flax plants under aluminum stress (Table 1: Conserved microRNAs; Table 2: MicroRNA expression; Table 3: MicroRNA targets).

## Figures and Tables

**Table 1:** Expression alterations of miRNAs after 4 and 24 hours of Al treatment in resistant (TMP1919 and G1071/4_k) and sensitive (G1071/4_o and Lira) flax cultivars and lines.

miRNA family	log_2_⁡FC									
	TMP1919		G1071/4_k		G1071/4_o		Lira		All samples	
	Al-4	Al-24	Al-4	Al-24	Al-4	Al-24	Al-4	Al-24	Al-4	Al-24
miR156	0.77	0.29	−1.79	−2.16	0.20	−1.58	0.74	−0.87	−0.42	−1.44
miR157	0.79	0.36	−1.40	−1.51	0.59	−1.93	−0.52	0.20	−0.26	−0.90
miR159	−2.81	−1.57	−0.11	−1.97	−0.51	−2.17	0.11	0.66	−0.62	−1.13
miR160	−0.23	−1.00	12.13	0.00	−0.30	−2.28	−1.22	−0.74	−0.35	−1.40
miR162	1.09	−0.86	−0.92	−0.29	−0.88	−1.94	−1.43	−1.78	−0.47	−1.04
miR164	1.27	−1.18	0.15	−1.72	0.50	−2.40	−2.49	0.46	0.61	−0.98
miR165	1.62	−0.84	−1.98	−3.16	0.19	−1.10	0.54	−1.86	−0.34	−2.13
miR166	1.13	−1.19	−1.75	−2.23	0.27	−1.60	−0.39	−2.33	−0.36	−1.98
miR167	−0.65	−1.46	−1.84	−15.07	−0.32	−2.34	−2.07	−0.22	−0.78	−1.54
miR168	0.20	−1.14	−0.39	−1.81	−0.30	−1.60	−0.60	−1.25	−0.28	−1.41
miR171	0.94	0.31	−2.36	−1.78	−1.22	−2.60	13.02	0.00	−0.01	−0.95
miR319	1.16	−0.65	0.22	−2.23	0.28	−2.04	0.45	−1.29	0.58	−1.40
miR390	1.27	−0.01	0.85	−0.84	−0.78	−3.04	−0.35	−1.77	0.17	−1.38
miR393	1.66	0.20	1.10	0.07	−0.46	−1.43	−0.10	0.51	0.61	−0.33
miR394	1.54	−0.18	13.71	12.33	−0.44	−1.99	−0.83	−1.24	0.71	−0.68
miR396	0.40	−1.45	−0.99	−1.90	−0.23	−1.51	−1.00	−1.26	−0.32	−1.45
miR398	−0.06	−1.22	0.00	0.00	0.38	−0.51	0.56	−2.83	0.47	−1.78
miR408	−0.17	−0.37	−0.35	−1.86	1.40	1.98	−2.20	−3.61	−0.82	−0.86
