# Erratum to “miR319, miR390, and miR393 Are Involved in Aluminum Response in Flax (*Linum usitatissimum* L.)”

**DOI:** 10.1155/2017/6915898

**Published:** 2017-08-13

**Authors:** Alexey A. Dmitriev, Anna V. Kudryavtseva, Nadezhda L. Bolsheva, Alexander V. Zyablitsin, Tatiana A. Rozhmina, Natalya V. Kishlyan, George S. Krasnov, Anna S. Speranskaya, Anastasia A. Krinitsina, Asiya F. Sadritdinova, Anastasiya V. Snezhkina, Maria S. Fedorova, Olga Yu. Yurkevich, Olga V. Muravenko, Maxim S. Belenikin, Nataliya V. Melnikova

**Affiliations:** ^1^Engelhardt Institute of Molecular Biology, Russian Academy of Sciences, Moscow 119991, Russia; ^2^All-Russian Research Institute for Flax, Torzhok 172002, Russia; ^3^Faculty of Biology, Lomonosov Moscow State University, Moscow 119991, Russia

In the article titled “miR319, miR390, and miR393 Are Involved in Aluminum Response in Flax (*Linum usitatissimum* L.)” [1], incorrect versions of Supplementary Table 2 “MicroRNA expression” and Supplementary Table 3 “MicroRNA targets” were published. The correct supplementary materials are available here.
