# Supplementary material for: miR319, miR390, and miR393 Are Involved in Aluminum Response in Flax (Linum usitatissimum L.)
Source: Biomed Res Int. 2017 Feb 19;2017:4975146. doi: 10.1155/2017/4975146 (PMC5337325; doi:10.1155/2017/4975146)
Supplement: Supplementary file 1 — MicroRNAs in flax plants under aluminum stress (Table 1: Conserved microRNAs; Table 2: MicroRNA expression; Table 3: MicroRNA targets). [file 4975146.f1.docx]

**Supplementary Table 1. MiRNAs from conserved families, which were identified in flax plants under aluminum stress.**

| **miRNA family** | **miRNA sequence** |
| --- | --- |
| miR156 | CUGACAGAAGAGAGUGAGCAC |
| miR156 | GCUCUCUAUGCUUCUGUCAUCA |
| miR156 | UGACAGAAGAGAGUGAGCAC |
| miR156 | UGACAGAAGAGAGUGAGCACU |
| miR156 | UUGACAGAAGAUAGAGAGC |
| miR156 | UUGACAGAAGAUAGAGAGCAC |
| miR156 | UUGACAGAAGAUAGAGAGCACA |
| miR156 | UUGACAGAAGAUAGAGAGCACC |
| miR156 | UUGACAGAAGAUAGAGAGCACU |
| miR156 | UUGACAGAAGAUAGAGAGCACUU |
| miR157 | GCUCUCUAUGCUUCUGUCAUC |
| miR157 | UUGACAGAAGAUAGAGAGCA |
| miR157 | UUGACAGAAGAUAGAGAGCACCU |
| miR159 | AUUGGAGUGAAGGGAGCUCGA |
| miR159 | UUGGAUUGAAGGGAGCUCU |
| miR159 | UUUGGAUUGAAGGGAGCU |
| miR159 | UUUGGAUUGAAGGGAGCUC |
| miR159 | UUUGGAUUGAAGGGAGCUCU |
| miR159 | UUUGGAUUGAAGGGAGCUCUC |
| miR159 | UUUGGAUUGAAGGGAGCUCUU |
| miR159 | UUUGGAUUGAAGGGAGCUCUUU |
| miR159 | UUUGGAUUGAAGGGAGCUCUUUU |
| miR159 | UUUGGAUUGAAGGGAGCUCUUUUU |
| miR160 | UGCCUGGCUCCCUGUAUGCCA |
| miR162 | UCGAUAAACCUCUGCAUCCA |
| miR162 | UCGAUAAACCUCUGCAUCCAG |
| miR164 | UGGAGAAGCAGGGCACGUGCA |
| miR165 | UCGGACCAGGCUUCAUCCCCC |
| miR166 | CCAGGCUUCAUUCCCC |
| miR166 | CGGACCAGGCUUCAUUCCCC |
| miR166 | CUCGGACCAGGCUUCAUUCC |
| miR166 | GGAAUGUUGUCUGGCUCGA |
| miR166 | GGAAUGUUGUCUGGCUCGAGG |
| miR166 | GGACCAGGCUUCAUUCCCC |
| miR166 | UCGGACCAGGCUUCAUU |
| miR166 | UCGGACCAGGCUUCAUUC |
| miR166 | UCGGACCAGGCUUCAUUCC |
| miR166 | UCGGACCAGGCUUCAUUCCC |
| miR166 | UCGGACCAGGCUUCAUUCCCC |
| miR166 | UCGGACCAGGCUUCAUUCCCCC |
| miR166 | UCGGACCAGGCUUCAUUCCCCU |
| miR166 | UCGGACCAGGCUUCAUUCCCU |
| miR166 | UCGGACCAGGCUUCAUUCCU |
| miR166 | UCGGACCAGGCUUCAUUCU |
| miR166 | UCUCGGACCAGGCUUCAUU |
| miR166 | UCUCGGACCAGGCUUCAUUC |
| miR166 | UCUCGGACCAGGCUUCAUUCCU |
| miR166 | UCUCGGACCAGGCUUCAUUCU |
| miR166 | UUGGACCAGGCUUCAUUCCCC |
| miR166 | UCUCGGACCAGGCUUUAUUCC |
| miR167 | UGAAGCUGCCAGCAUGAUCUG |
| miR167 | UGAAGCUGCCAGCAUGAUCUGG |
| miR168 | CCCGCCUUGCAUCAACUGAAU |
| miR168 | UCGCUUGGUGCAGGUCGGGA |
| miR168 | UCGCUUGGUGCAGGUCGGGAA |
| miR168 | UCGCUUGGUGCAGGUCGGGAAC |
| miR168 | UCGCUUGGUGCAGGUCGGGAAU |
| miR168 | UCGCUUGGUGCAGGUCGGGAU |
| miR171 | UUGAGCCGCGCCAAUAUCACU |
| miR171 | UGAGCCGAACCAAUAUCACUC |
| miR319 | CUUGGACUGAAGGGAGCUCC |
| miR319 | CUUGGACUGAAGGGAGCUCCC |
| miR319 | CUUGGACUGAAGGGAGCUCCCC |
| miR319 | CUUGGACUGAAGGGAGCUCCCU |
| miR319 | CUUGGACUGAAGGGAGCUCCCUU |
| miR319 | CUUGGACUGAAGGGAGCUCCCUUUU |
| miR319 | UUGGACUGAAGGGAGCUCC |
| miR319 | UUGGACUGAAGGGAGCUCCC |
| miR319 | UUGGACUGAAGGGAGCUCCCC |
| miR319 | UUGGACUGAAGGGAGCUCCCU |
| miR319 | UUGGACUGAAGGGAGCUCCCUU |
| miR319 | UUGGACUGAAGGGAGCUCCCUUU |
| miR319 | UUGGACUGAAGGGAGCUCCCUUUU |
| miR319 | UUGGACUGAAGGGAGCUCCUU |
| miR390 | AAGCUCAGGAGGGAUAGCGCC |
| miR390 | AAGCUCAGGAGGGAUAGCGCCU |
| miR390 | CGCUAUCCAUCCUGAGUUUCC |
| miR393 | UCCAAAGGGAUCGCAUUGAUC |
| miR393 | UCCAAAGGGAUCGCAUUGAUCU |
| miR393 | UCCAAAGGGAUCGCAUUGAUU |
| miR393 | UCCAAAGGGAUCGCAUUGAUUU |
| miR393 | UCCAAAGGGAUCGCAUUGAUUUU |
| miR394 | UUGGCAUUCUGUCCACCUCC |
| miR396 | ACAGCUUUCUUGAACUG |
| miR396 | CACAGCUUUCUUGAACUG |
| miR396 | CAGCUUUCUUGAACUG |
| miR396 | CUCAAGAAAGCUGUGGGAGA |
| miR396 | GUUCAAUAAAGCUGUGGGAAG |
| miR396 | UCCACAGCUUUCUUGAACUG |
| miR396 | UUCCACAGCUUUCUUGAAC |
| miR396 | UUCCACAGCUUUCUUGAACU |
| miR396 | UUCCACAGCUUUCUUGAACUG |
| miR396 | UUCCACAGCUUUCUUGAACUU |
| miR398 | UGUGUUCUCAGGUCGCCCCUG |
| miR408 | AUGCACUGCCUCUUCCCUGGCC |
| miR408 | UGCACUGCCUCUUCCCUGGCC |
| miR408 | UGCACUGCCUCUUCCCUGGCCU |
