# Supplementary material for: miR319, miR390, and miR393 Are Involved in Aluminum Response in Flax (Linum usitatissimum L.)
Source: Biomed Res Int. 2017 Feb 19;2017:4975146. doi: 10.1155/2017/4975146 (PMC5337325; doi:10.1155/2017/4975146)
Supplement: Supplementary file 2 [file 4975146.f2.docx]

**Supplementary Table 2. Expression of miRNA families in flax cultivars and lines ‘TMP1919’, ‘G1071/4_k’, ‘G1071/4_o’, and ‘Lira’.**

| **miRNA family** | **Read per million** | | | | | | | | | | | |
| --- | --- | --- | --- | --- | --- | --- | --- | --- | --- | --- | --- | --- |
|  | **TMP1919** | | | **G1071/4_k** | | | **G1071/4_o** | | | **Lira** | | |
|  | **N** | **Al-4** | **Al-24** | **N** | **Al-4** | **Al-24** | **N** | **Al-4** | **Al-24** | **N** | **Al-4** | **Al-24** |
| **miR156** | 736 | 1252 | 903 | 5237 | 1509 | 1175 | 1784 | 2053 | 596 | 1082 | 1807 | 594 |
| **miR157** | 58 | 100 | 74 | 291 | 110 | 102 | 136 | 204 | 36 | 77 | 53 | 88 |
| **miR159** | 4847 | 692 | 1638 | 256 | 238 | 65 | 14625 | 10304 | 3254 | 3639 | 3919 | 5735 |
| **miR160** | 12 | 10 | 6 | 0 | 4 | 0 | 31 | 25 | 6 | 18 | 8 | 11 |
| **miR162** | 93 | 198 | 51 | 194 | 102 | 159 | 146 | 80 | 38 | 193 | 72 | 56 |
| **miR164** | 46 | 111 | 20 | 22 | 24 | 7 | 33 | 46 | 6 | 21 | 4 | 29 |
| **miR165** | 9 | 28 | 5 | 119 | 30 | 13 | 37 | 42 | 17 | 45 | 65 | 12 |
| **miR166** | 10367 | 22724 | 4556 | 42385 | 12617 | 9019 | 13844 | 16688 | 4553 | 22667 | 17327 | 4521 |
| **miR167** | 49 | 31 | 18 | 34 | 10 | 0 | 129 | 103 | 25 | 57 | 14 | 49 |
| **miR168** | 536 | 613 | 244 | 459 | 352 | 131 | 802 | 651 | 265 | 853 | 562 | 360 |
| **miR319** | 2948 | 6582 | 1879 | 2200 | 2568 | 468 | 3897 | 4735 | 945 | 4617 | 6328 | 1893 |
| **miR390** | 98 | 235 | 97 | 84 | 153 | 47 | 211 | 123 | 26 | 199 | 156 | 58 |
| **miR393** | 18 | 56 | 20 | 53 | 114 | 56 | 68 | 49 | 25 | 14 | 13 | 19 |
| **miR394** | 5 | 15 | 5 | 0 | 13 | 5 | 14 | 10 | 3 | 7 | 4 | 3 |
| **miR396** | 1688 | 2220 | 617 | 922 | 464 | 247 | 1264 | 1080 | 444 | 2186 | 1093 | 910 |
| **miR398** | 4 | 4 | 2 | 0 | 0 | 0 | 10 | 13 | 7 | 31 | 46 | 4 |
| **miR408** | 18 | 16 | 14 | 56 | 44 | 15 | 58 | 154 | 229 | 397 | 87 | 33 |
